# Toward trustworthy artificial intelligence in multi-omics: a review of reproducibility, stability, and interpretability

**DOI:** 10.1093/bib/bbag227

**Published:** 2026-05-11

**Authors:** Thanh Hoa Vo, Nguyen Quoc Khanh Le

**Affiliations:** Department of Science, South East Technological University, Cork Road, Waterford City, Co. Waterford, X91 K0EK, Ireland; Pharmaceutical and Molecular Biotechnology Research Center (PMBRC), Cork Road, Waterford City, Co. Waterford, X91 K0EK, Ireland; AIBioMed Research Group, Taipei Medical University, No. 250 Wuxing St., Xinyi Dist., Taipei 110, Taiwan; AIBioMed Research Group, Taipei Medical University, No. 250 Wuxing St., Xinyi Dist., Taipei 110, Taiwan; In-Service Master Program in Artificial Intelligence in Medicine, College of Medicine, Taipei Medical University, No. 250 Wuxing St., Xinyi Dist., Taipei 110, Taiwan; Translational Imaging Research Center, Taipei Medical University Hospital, No. 252 Wuxing St., Xinyi Dist., Taipei 110, Taiwan

**Keywords:** multi-omics, trustworthy AI, reproducibility, stability, interpretability, precision medicine

## Abstract

The integration of multi-omics data has become increasingly important in advancing precision medicine and systems biology. However, the reliability and trustworthiness of artificial intelligence (AI) models applied to such data remain critical concerns. This review examines the evolution and current landscape of reproducibility, stability, and interpretability in AI-driven multi-omics analysis. We explore these three pillars of trustworthiness in recent literature, with a particular focus on methodological innovations, benchmarking practices, and biological relevance. Drawing from key publications, including those featured in *Briefings in Bioinformatics*, we highlight emerging frameworks that aim to make multi-omics models more robust, transparent, and translationally meaningful. We advocate for routine adoption of TRUST-aligned evaluation practices, including structured stability assessments, multi-cohort benchmarking, and standardized model-card reporting, as default components of future multi-omics AI development. We conclude by outlining key challenges and future directions for developing trustworthy AI systems capable of supporting reproducible, interpretable, and clinically meaningful multi-omics research.

## Introduction

Over the past 25 years, advances in multi-omics technologies and artificial intelligence (AI) have transformed how complex biological systems are analyzed and interpreted. Early computational studies focused on single-omics measurements, but innovations in sequencing, mass spectrometry, and high-throughput profiling expanded the field toward integrated, multi-layer molecular characterization. In parallel, machine learning and deep learning methods matured into central analytical tools capable of modeling high-dimensional omics data and capturing regulatory relationships across modalities [1–7]. Recent comprehensive surveys of deep learning-driven multi-omics integration further highlight both rapid methodological innovation and the growing complexity of model architectures and evaluation practices [8, 9]. Additionally, current methodological developments in AI-driven anticancer compound screening and computational drug design, including attention-based architectures and generative modeling frameworks, further demonstrate how advances in representation learning and performance assessment are reshaping biomedical prediction tasks beyond omics integration alone [10, 11]. Building on this trajectory, this review introduces the TRUST framework, a structured perspective for evaluating trustworthy AI in multi-omics.

**Table 1:** Categories of interpretability methods in multi-omics AI.

Category	Description	Representative examples
**Intrinsic interpretability**	Models that are transparent by design and provide human-readable decision rules.	Sparse linear models, decision trees, rule-based ensembles, patient-similarity networks [32, 33].
**Post-hoc interpretability**	Methods applied after model training to explain complex models locally or globally.	SHAP, LIME [1, 2]; saliency maps, perturbation analyses [34–37]; multi-omics applications such as DeepProg and MOGONET [38].
**Architecture-guided interpretability**	Models that embed biological structure or mechanisms to produce interpretable outputs.	Attention-based multimodal models [3]; GNNs with GNNExplainer [4]; generative models such as scMM [5]; factor models MOFA and MOFA+ [6, 7].

**Table 2:** Sources of instability in multi-omics AI and representative mitigation strategies.

Source of instability	Why it occurs	Impact on models	Representative methods or approaches
**Heterogeneous noise across omics layers**	Each modality has distinct variance, sparsity, biases, and batch effects [40–42].	Unstable feature weights, shifted latent spaces, inconsistent predictions	Batch correction, modality-specific preprocessing, noise-aware fusion
**Modality-specific missingness and uneven coverage**	Proteomics, metabolomics and spatial omics often contain missing or low-coverage signals [40–42].	Model sensitivity to weak layers, unpredictable behavior when modalities are incomplete	Hybrid or attention-based fusion [43, 46–48]; cross-modality generation with VAEs or diffusion models [52–54]
**Small or imbalanced sample composition**	Many multi-omics datasets have modest sample sizes or uneven populations [1, 2].	High variance in training results, inconsistent clustering, or risk groups	Subsampling, resampling, stability selection
**Architecture-dependent variability**	Early fusion, late fusion, and hybrid architectures respond differently to noise or missing data [43–48].	Variation in learned representations and cross-layer interactions	Structured fusion strategies; biological priors such as PPI or pathway graphs [49–51]
**Sensitivity to parameter initialization and training conditions**	Deep models in high-dimensional spaces depend strongly on optimization dynamics [14–16].	Different runs produce different feature rankings or clusters	Repeated training, perturbation testing, regularization strategies
**Limited stability evaluation practices**	Most studies focus on accuracy, not robustness [55–58].	Unclear reliability of selected biomarkers, or patient stratification	Nogueira stability score [55]; StabilityCCA [56]; Stabl framework [57]

Multi-omics refers to the integrated analysis of two or more molecular data types derived from the same or related biological systems. Integration can take several forms. Vertical integration combines multiple omics layers within the same samples, such as joint modeling of transcriptomics, epigenomics, and proteomics. Horizontal integration links datasets across different cohorts or studies measuring similar modalities. Multimodal integration encompasses complex data combinations such as proteogenomics, spatial transcriptomics, and single-cell multi-omics, which require models capable of capturing relationships across distinct measurement platforms [3–7, 12, 13]. These strategies allow multi-omics AI models to characterize cellular states more comprehensively than single-omics approaches.

**Figure 1:**
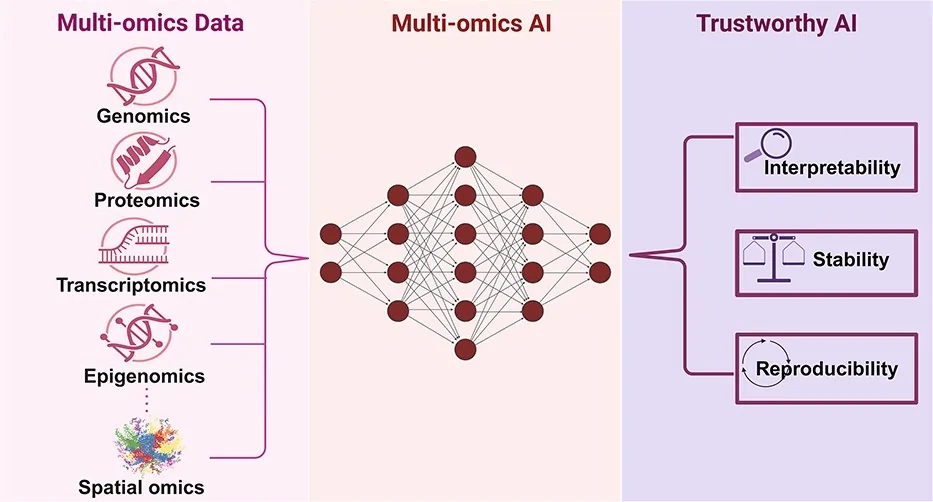
Conceptual illustration of multi-omics data integration and its relationship to the core dimensions of trustworthy AI. Multiple omics layers, including genomics, epigenomics, transcriptomics, proteomics, and spatial or single-cell data, are integrated using machine learning and deep learning models that capture cross-layer regulatory relationships. The three pillars examined in this review, interpretability, stability, and reproducibility, represent key criteria for evaluating the reliability and biological grounding of multi-omics AI models.

As multi-omics AI has expanded, concerns regarding transparency, robustness, and reproducibility have become increasingly prominent. Multi-omics datasets exhibit heterogeneity, batch effects, and modality-specific noise, all of which can strongly influence model behavior and contribute to instability or irreproducible results [14–18]. Long-standing data standards such as MIAME and MINSEQE [19, 20], as well as FAIR principles for data stewardship [21], highlight the importance of consistent documentation and structured reporting, yet their adoption in AI workflows remains uneven. Recent benchmark studies further demonstrate that model performance often varies substantially across datasets and cancer types, reinforcing the need for rigorous cross-cohort evaluation and unified assessment frameworks [22–25]. Our bibliometric analysis reflects these developments, showing a marked rise in publications addressing interpretability, stability, and reproducibility, especially after 2020. Key acronyms and methodological terms used throughout the manuscript are summarized in Supplementary Table S1. The studies and methodological examples summarized in Tables 1 and 2 were selected to illustrate representative categories of interpretability and stability approaches in multi-omics AI rather than to provide an exhaustive systematic review. Included works primarily reflect recent methodological developments published in leading bioinformatics and computational biology journals, with oncology-focused applications proportionally represented due to the high volume of multi-omics integration research in cancer. A conceptual illustration of the flow from multi-omics data to AI modeling and the trustworthiness dimensions that motivate this review is presented in Fig. 1.

## Bibliometric overview of AI and multi-omics research

To examine the development of AI-driven multi-omics research, we conducted a structured bibliometric analysis using two complementary datasets: (i) publications in *Briefings in Bioinformatics* (*BIB*) between 2000 and 2025, and (ii) a global reference dataset retrieved from Scopus using the same AI and multi-omics search vocabulary across all journals. Metadata were downloaded on 22 November 2025. Records were restricted to articles and reviews published between 2000 and 2025.

Titles, abstracts, author keywords, and index keywords were combined into a unified text field and processed using a standardized text analysis workflow. AI-related publications were identified using the following predefined terms: ‘machine learning’, ‘deep learning’, ‘artificial intelligence’, ‘neural network’, ‘graph neural network’, ‘gnn’, ‘random forest’, ‘support vector machine’, ‘svm’, ‘autoencoder’, ‘representation learning’, and ‘transformer’. Multi-omics publications were identified using explicit terminology including: ‘multi-omics’, ‘multiomics’, ‘multi omics’, ‘omics integration’, ‘proteogenomics’, ‘spatial omics’, ‘single cell multi omics’, ‘pan-omics’, ‘pan omics’, ‘multi-modal’, and ‘multi modal’. In addition to explicit terminology, a heuristic definition classified records as multi-omics when at least two distinct omics layers were co-mentioned within the same publication. AI + multi-omics publications were defined as records satisfying both criteria. A sensitivity analysis comparing explicit-only and heuristic-expanded definitions was performed to evaluate classification robustness.

Annual publication counts were calculated using calendar-year bins based on the Scopus ‘Year’ field and are reported as raw counts. The global dataset provides a field-level benchmark, while the *BIB* dataset offers a focused view of developments within a leading bioinformatics journal.

**Figure 2:**
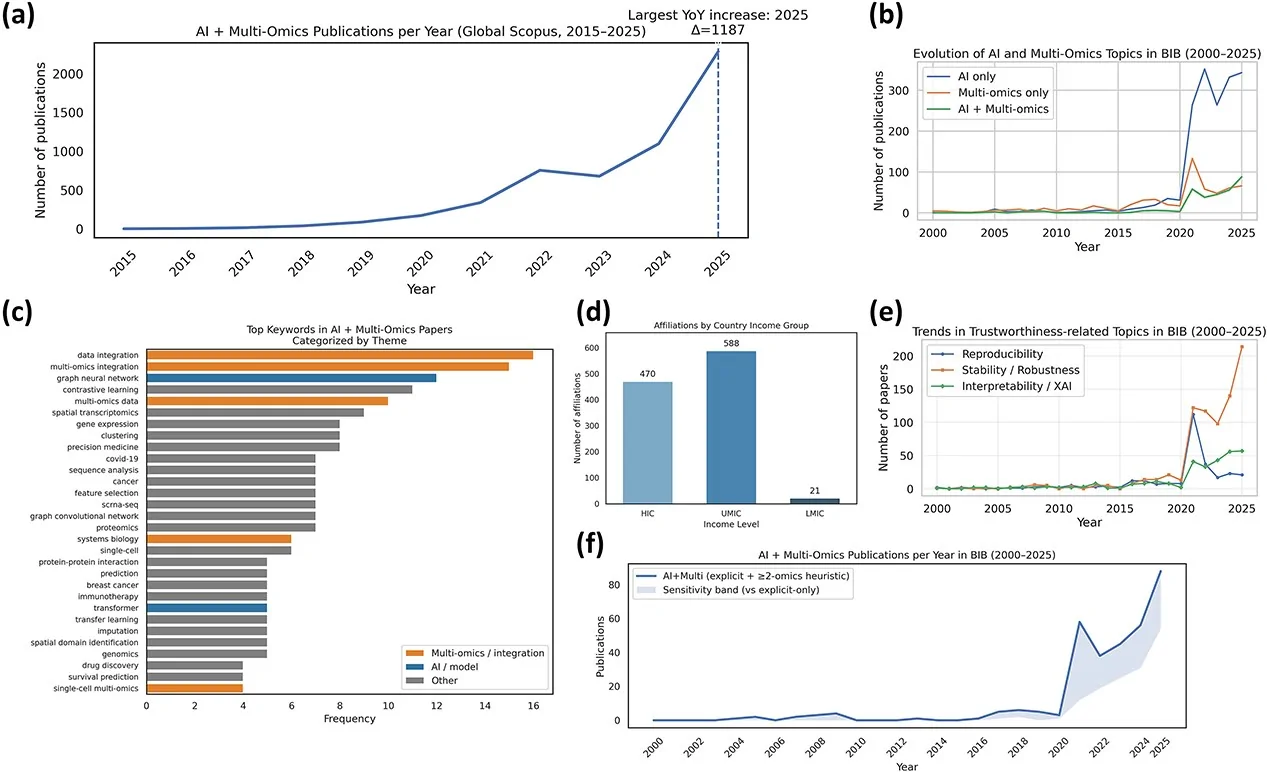
Bibliometric landscape of AI-driven multi-omics research. (a) Global annual counts of AI + multi-omics publications indexed in Scopus (2015–2025); the dashed line marks the largest year-over-year increase. (b) Annual distribution of AI-only, multi-omics-only, and AI + multi-omics articles in *BIB* (2000–2025); the shaded area reflects differences between operational definitions. (c) Most frequent thematic keywords in AI + multi-omics publications in *BIB*. (d) Author affiliations by World Bank income group. (e) Trends in trustworthiness-related themes (reproducibility, robustness/stability, interpretability/XAI) in *BIB* (2000–2025). (f) Annual AI + multi-omics publications in *BIB* with sensitivity analysis (explicit vs. expanded heuristic definition).

The global analysis reveals limited activity prior to 2015, followed by sustained growth and a sharp acceleration after 2020 (Fig. 2a). The most pronounced year-over-year increase occurs in the most recent indexing years, reflecting rapid expansion of AI-enabled multi-omics methodologies across disciplines. Trends observed within *BIB* (Fig. 2b and f) closely parallel these broader dynamics, with a marked increase in AI + multi-omics publications after 2020. The sensitivity analysis indicates that a substantial fraction of recent publications integrates multiple omics layers without explicitly using the term ‘multi-omics’, underscoring the importance of operational definitions in bibliometric classification.

Thematic analysis of keywords across AI + multi-omics publications highlights a strong emphasis on integrative modeling and advanced learning architectures, including graph neural networks, transformer-based models, contrastive learning, and spatial transcriptomics (Fig. 2c). Over time, there is also an increasing presence of terms related to reproducibility, robustness, and interpretability (Fig. 2e), suggesting growing attention to methodological reliability and transparent model design.

Geographic analysis, based on author affiliations mapped to World Bank income group classifications (World Bank income group dataset, accessed 22 November 2025), indicates that contributions are predominantly concentrated in high-income and upper middle-income regions (Fig. 2d). Representation from lower middle-income regions remains comparatively limited, reflecting disparities in computational infrastructure, large-scale data access, and specialized training capacity.

Together, the global- and journal-level analyses demonstrate that AI-enabled multi-omics research has transitioned from an emerging methodological niche to a rapidly expanding and structurally maturing field. These temporal, thematic, and geographic patterns provide essential context for the methodological challenges discussed in the following sections.

## Interpretability in AI models for multi-omics

Interpretability is an essential component of trustworthy AI in multi-omics analysis. In machine learning, interpretability refers to the degree to which a model can be understood by a human, whereas explainability describes the contribution of specific features to a prediction [26–28]. In this review, we use ‘interpretability’ as an umbrella term encompassing both intrinsically transparent models and explanation techniques applied to complex models. In biomedical contexts, interpretability requires that a model reveal the decision criteria that connect molecular inputs to biological or clinical outputs rather than producing an opaque probability score. Limited transparency remains a major barrier to adoption in translational settings even when predictive performance is high [29–31].

Interpretability methods used in multi-omics research fall into three broad categories. First, intrinsically interpretable models are transparent by design. Sparse linear models, decision trees, and rule-based ensembles provide explicit decision paths that clinicians can inspect directly [32]. Patient-similarity networks, which integrate heterogeneous molecular and clinical data to represent patients as nodes linked by biological similarity, further enable transparent visual inspection of disease subgroups and molecular clusters [33].

Second, post-hoc model explanations are applied to complex deep learning models. Popular approaches include SHAP and LIME, which provide locally faithful approximations of model behavior [1, 2]. Gradient-based saliency maps and perturbation-based methods reveal which genomic regions or omics features most strongly influence predictions [34, 35]. These approaches have been widely used to interpret regulatory sequence models, where convolutional filters and saliency patterns reveal transcription factor motifs and regulatory logic [36, 37]. In multi-omics analyses, post-hoc interpretability methods such as SHAP and gradient-based saliency have been applied to quantify how transcriptomic, DNA methylation, and other epigenomic features contribute to predictive performance, as demonstrated in models like DeepProg and MOGONET [15, 38].

Third, architecture-guided interpretability methods leverage biological structure that is explicitly encoded into multi-omics models. Attention mechanisms in multimodal deep learning frameworks highlight informative genes, chromatin regions or pathways by weighting modality-specific, or feature-level contributions during prediction [3]. Multi-omics graph neural networks integrate gene, protein, or cell–cell interaction graphs and can be interrogated using graph-explanation tools such as GNNExplainer to identify influential nodes, edges, or pathways underlying model outputs [4]. Deep generative models designed for multi-omics, such as scMM, provide interpretable latent dimensions that capture coordinated regulatory programs across transcriptomic and chromatin layers at cellular resolution [5]. Finally, factor-analysis frameworks such as MOFA and MOFA+ learn biologically meaningful low-dimensional factors that summarize shared and modality-specific variation across transcriptomic, epigenomic, and proteomic layers [6, 7].

These interpretability strategies have yielded mechanistic insights in multi-omics studies. Saliency and perturbation methods have revealed motif-level regulatory patterns that influence predictions across integrated omics data [36]. Pathway-informed neural architectures compress complex multi-omics signatures into interpretable pathway level scores [39]. Latent factor models have identified shared regulatory axes that connect variation across multiple omics modalities [6]. While the methods above describe how interpretability is implemented, systematic evaluation should also consider biological validity and clinical relevance. Biological significance requires that attributed features, pathways, or latent factors correspond to established regulatory mechanisms or disease-associated processes rather than dataset-specific artefacts, an issue widely discussed in interpretable machine learning and genomics applications [26–28]. Validation may involve pathway enrichment analysis, replication across independent cohorts, and concordance with experimentally validated biomarkers. Clinical relevance emerges when model explanations align with prognostic signatures, therapeutic targets, or subtype-defining molecular programs that inform patient stratification, thereby supporting responsible clinical deployment of AI systems [29, 31]. Importantly, prior work has shown that attribution methods can be sensitive to correlated features or training variability, emphasizing the need for robustness checks before biological or clinical interpretation [27, 28]. Table 1 provides a concise overview of the primary categories of interpretability methods in multi-omics AI.

## Stability in multi-omics AI models

While reproducibility concerns whether identical computational conditions yield identical outputs, stability evaluates whether model behavior remains consistent under controlled perturbations of data, sampling, or initialization. Stability represents a fundamental pillar of trustworthy AI in multi-omics, referring to the extent to which a model produces consistent outputs when confronted with minor, non-biological perturbations such as small variations in sample composition, preprocessing procedures, or parameter initialization. In this context, we define uniform stability as the requirement that such consistency is maintained across repeated training and structured perturbation analyses. Deep learning models operating in high-dimensional multi-omics settings are particularly sensitive to these perturbations due to the heterogeneous noise profiles, batch effects, and sparsity patterns inherent to each molecular layer [14]. These challenges are further amplified in single-cell multi-omics datasets, where dropout events, cell-level heterogeneity, and cross-modality alignment variability can substantially influence clustering, trajectory inference, and downstream interpretation [5, 7]. This sensitivity is well documented in recent deep learning roadmaps and multi-omics integration studies, which show that even modest adjustments to input distributions or fusion strategies can alter learned representations, impact patient stratification results, and shift biomarker rankings [14–16]. Taken together, these studies indicate that high performance in a single evaluation does not necessarily imply stable behavior, and that such instability can obscure biological signals and limit the translational reliability of multi-omics AI systems [17, 18].

Many sources of instability in multi-omics AI originate from the statistical characteristics of the underlying data. Different molecular layers are known to exhibit distinct noise profiles and technical variability: transcriptomic measurements often show high biological and technical variance, DNA methylation can be affected by platform-specific biases and batch structure, and proteomic or metabolomic assays frequently contain missing values, sparsity, or inconsistent detection sensitivity [40, 41]. These modality-specific differences are widely documented in multi-omics integration studies as major challenges for data harmonization and fusion, particularly due to heterogeneity across data types, high dimensionality, and incomplete modality coverage [40–42]. Spatial and other multi-modal omics technologies introduce further variability through differences in sequencing depth, coverage, and assay protocols, which can influence downstream analyses such as clustering or spatial domain identification [12, 13]. In addition, many multi-omics datasets have modest sample sizes or uneven population representation, contributing to variability during model training and validation [1, 2]. These characteristics highlight that data heterogeneity, missingness, and modality-specific variability are important considerations when evaluating stability in multi-omics AI models.

Model architecture plays an important role in determining how multi-omics AI models respond to heterogeneous and incomplete data. Early approaches often relied on simple feature concatenation across omics layers, which combines all modalities at the input level and can be affected by differences in scale, distribution, or missingness between data types [43–45]. As multi-omics datasets frequently vary in quality across modalities, more recent architectures introduce structured fusion strategies, including early fusion, late fusion, hybrid fusion, and attention-based fusion, to better model modality-specific contributions and reduce the impact of noisy or weak layers [43, 46–48]. Another major direction incorporates biological priors into architecture. Models that use protein–protein interaction networks, gene-regulatory graphs, or curated pathway structures apply these priors to guide representation learning and encourage biologically meaningful patterns [49–51]. A further group of methods focuses on cross-modality translation and completion, including cross-omics variational autoencoders, multimodal autoencoders, and diffusion-based generative models that learn shared latent spaces or mappings to reconstruct or infer missing omics layers [52–54]. These generative approaches evaluate performance through reconstruction accuracy, agreement between predicted and observed modalities, and the quality of cross-modality alignment under different input conditions [52–54].

Explicit evaluation of stability remains uncommon in the current multi-omics AI literature, where most studies continue to prioritize predictive accuracy as the main performance measure. When stability is examined, it is typically assessed at the level of feature or biomarker selection rather than at the level of the full model. Several recent analyses illustrate this trend. For example, Łukaszuk et al. used the Nogueira stability metric to evaluate L1-regularized classifiers across multiple The Cancer Genome Atlas (TCGA) cancer types and showed that selected features can vary considerably with regularization strength, with different omics layers exhibiting distinct stability profiles [55]. Pusa and Rousu introduced StabilityCCA, demonstrating that applying stability selection to sparse canonical correlation analysis can identify variables that remain consistently selected across resampled datasets [56]. The Stabl framework adopts a similar strategy by incorporating noise injection and a data-driven signal-to-noise threshold, and its evaluation across synthetic data and five independent clinical studies, including multi-omic integration tasks, reports improved sparsity, and reproducibility of selected biomarkers compared with standard regularization approaches [57]. Recent reviews of deep learning with multi-omics data also note that, despite rapid methodological progress, systematic benchmarking, and robustness assessments remain limited, and they argue that stability and reliability should be treated as explicit evaluation criteria rather than assumed to follow from high accuracy alone [58].

Building upon stability analyses and perturbation-based approaches reported in recent multi-omics studies [14–16], stability in multi-omics AI should be evaluated using a structured perturbation battery and applied systematically during model development. At minimum, models should be retrained across multiple random seeds (e.g. ≥10 independent initializations) to quantify variability in predictive metrics, feature rankings, and cluster assignments. Sampling variability should be assessed using bootstrap resampling or repeated subsampling (e.g. 80% cohort resampling across ≥20 iterations), with dispersion in performance, Jaccard similarity of selected biomarkers and adjusted Rand index (ARI) for clustering reported. Given the heterogeneous structure of multi-omics data, modality-specific stress testing is also recommended, including systematic modality dropout and feature masking at graded missingness levels (e.g. 5%–20%) to evaluate robustness to incomplete layers. Controlled perturbations such as Gaussian noise injection proportional to feature variance or synthetic batch shifts can further quantify tolerance to non-biological technical variation. Stability reporting should extend beyond predictive accuracy and include dispersion metrics for predictions (standard deviation across runs), feature importance consistency (rank correlations or overlap indices), clustering agreement (ARI or Normalized Mutual Information (NMI)), and latent representation alignment where applicable. Such structured evaluation enables stability to be assessed explicitly rather than inferred indirectly from single-run performance.

Stability remains an underexamined aspect of multi-omics AI, and most assessments focus on how selected features change under different modeling choices. Existing studies show that multi-omics models can be sensitive to data heterogeneity, modality imbalance, and architectural decisions, which can influence the consistency of their outputs. Although systematic evaluation is still limited, the available evidence suggests that variability in model behavior has important implications for how biological results are interpreted. A clearer picture of these effects will remain important as multi-omics AI continue to expand. A concise summary of the main sources of instability in multi-omics AI and representative methods used to assess or mitigate them is provided in Table 2.

## Reproducibility in AI for multi-omics

Reproducibility refers to the ability to regenerate results under identical computational and data conditions and is distinct from stability, which evaluates robustness to perturbations. In multi-omics AI, reproducibility is a core requirement of trustworthy analysis and encompasses the ability to re-execute the same pipeline using the same data, code, software versions, and analytical settings to obtain identical outcomes [59, 60]. Multi omics research faces particular challenges because datasets often differ in preprocessing conventions, cohort structure, and measurement conditions, which can influence downstream model behavior [23]. Foundational community standards such as MIAME, MINSEQE, and the MAQC projects established the importance of clear reporting and cross platform validation for generating reliable molecular profiles [19, 20]. More recent initiatives, including the FAIR principles, extend these expectations to data stewardship and computational documentation [21]. Workflow engines and containerized environments provide additional strategies for ensuring that software dependencies and execution conditions remain stable across analyses [61, 62]. Survey studies in bioinformatics consistently highlight reproducibility as a key requirement for building trustworthy multi-omics AI systems, particularly given the strong dataset dependence observed across many current models [23, 63].

Benchmarking has become one of the most effective practical strategies for improving reproducibility in multi-omics AI. Recent studies have introduced standardized evaluation frameworks that compare models across shared datasets, unified metrics, and controlled pipelines, helping to reduce variability arising from inconsistent analytical choices [22, 23]. Large-scale benchmarks in survival prediction and integrative classification demonstrate that model performance varies substantially across datasets and cancer types, indicating that reproducibility cannot be inferred from results obtained on a single cohort [23–25]. Similar trends are observed in domain specific benchmarking efforts, including spatial transcriptomics clustering, transcription factor binding prediction, and radiosensitivity signature analysis, where systematic comparisons reveal strong dataset dependence and highlight the need for multi dataset evaluation to obtain reliable conclusions [64–67]. Survey studies in bioinformatics further emphasize that such benchmark driven evaluation is essential for establishing reproducible methodological baselines and for enabling fair comparison across emerging multi-omics AI models [63]. Together, these efforts show that reproducibility in multi-omics AI increasingly relies on transparent benchmarking practices and evaluation across heterogeneous datasets.

Reproducibility in multi-omics AI is further constrained by variation across cohorts, platforms, and molecular layers. In single-cell multi-omics studies, reproducibility further depends on consistent preprocessing choices, including quality control thresholds, normalization strategies, cell-type annotation procedures, and modality alignment algorithms. Differences in these steps can lead to divergent clustering structures and latent embeddings even when using the same raw datasets, highlighting the importance of standardized single-cell workflows and transparent reporting of integration parameters [5, 7, 53, 54]. Multi-cancer benchmark studies demonstrate that models trained on one cohort often show reduced performance on others, indicating that cohort specific characteristics can strongly influence predictive behavior [23–25]. Reviews of multi-omics integration also note that heterogeneity in assay platforms, sample composition, and preprocessing pipelines introduces shifts in data distributions that complicate model transfer across studies [68, 69]. Frameworks such as ImmuneMirror and multi-omics generative models report that latent representations and feature contributions can vary across datasets, reinforcing the need for external validation when assessing model reliability [70, 71]. A bioinformatics survey underscores that cross-cohort sensitivity hinders reproducible multi-omics AI, and that independent dataset validation is critical for generalizability [63].

Reproducibility in multi-omics AI also depends on the clarity and consistency of reporting practices. Early standards such as MIAME, MINSEQE, and the MAQC initiatives demonstrated that transparent documentation of assay design, metadata, and processing steps is essential for generating reliable molecular measurements [19, 20, 72]. More recent frameworks, including the FAIR principles, extend these expectations to data accessibility, interoperability, and reuse, which are increasingly relevant as multi-omics workflows become more computationally complex [21]. Reproducible pipelines also benefit from workflow engines and containerized environments that preserve software dependencies and execution conditions, providing stability across platforms and analytical runs [61, 62, 73]. Although these practices are not yet universally adopted in multi-omics AI, several benchmark studies have begun to incorporate standardized pipelines and unified evaluation frameworks, reflecting broader recognition that reproducible workflows are necessary for trustworthy model development [22, 23]. An article in bioinformatics similarly emphasize that improved documentation and transparent computational reporting remain central to advancing reproducibility in the field [74].

Although reproducibility has received increasing attention in multi-omics AI, existing practices remain uneven. Benchmark studies illustrate that reproducible performance depends strongly on transparent pipelines and evaluation across heterogeneous datasets, while cross-cohort analyses show that multi-omics variability can significantly influence model behavior [22–24]. Standards such as MIAME, MINSEQE, and FAIR provide clear guidance for data reporting and documentation, yet their implementation across computational workflows is still inconsistent [19, 21]. Together, these observations indicate that reproducibility in multi-omics AI requires broader adoption of structured reporting, reproducible pipelines, and multi-dataset validation. Continued progress in these areas will be essential for establishing reliable, trustworthy AI systems that can be deployed with confidence in biological and clinical settings. A summary of the major factors affecting reproducibility in multi-omics AI and the strategies used to address them is provided in Table 3.

**Table 3:** Determinants of reproducibility in multi-omics AI and corresponding evaluation practices.

Determinant of reproducibility	How it affects multi-omics AI	Representative strategies or frameworks
**Preprocessing and cohort variability**	Differences in preprocessing, cohort structure, and measurement conditions change model behavior [23].	Transparent documentation; standardized pipelines; early reporting standards (MIAME, MINSEQE, MAQC) [19, 20].
**Data stewardship and metadata quality**	Lack of consistent reporting hinders regeneration of analyses and model transfer [19, 21].	FAIR principles for accessibility, interoperability, and reuse [21].
**Computational environment reproducibility**	Software dependencies, versions, and execution conditions affect reproducibility across platforms [61, 62].	Workflow managers (Nextflow, Snakemake); containerization (Docker, Singularity) [61, 62].
**Dataset dependence of model performance**	Strong variation across datasets, cancer types, and modalities limits reproducibility [22–24, 64–67].	Multi-dataset benchmarking; unified metrics; cross-cohort evaluation [22–24, 64–67].
**Cross-cohort variability**	Models trained on one cohort often show reduced performance on others due to platform and composition differences [23–25, 68, 69].	External validation; evaluation across independent cohorts; multi-cohort generative models (e.g. ImmuneMirror) [70, 71].
**Insufficient reporting of computational details**	Missing workflow documentation reduces reproducibility and comparability across studies [72–74].	Improved reporting of code, metadata and assumptions; adoption of reproducible workflow standards [72–74].

## Toward a unified framework for trustworthy AI in multi-omics

Recent trends observed in our bibliometric analysis show that research in *BIB* has increasingly engaged with concepts related to interpretability, stability, and reproducibility over the past decade. The rise of terms such as model transparency, robustness, benchmark evaluation, and multi-omics integration after 2020 reflects a broader shift toward AI systems that are not only accurate but also reliable and biologically grounded. Although progress has been considerable, our review indicates that these three dimensions are often examined independently and that methodological practices remain inconsistent across studies. These observations motivate the need for a unified conceptual framework to guide the development and evaluation of trustworthy multi-omics AI systems.

Gaps identified across the preceding sections highlight complementary areas that require systematic alignment. Interpretability research spans intrinsically interpretable models [32], post-hoc explanation methods [1, 2], and architecture-guided strategies [3–7], yet standard evaluation procedures and biological validation remain uneven. Stability is rarely assessed explicitly, despite extensive evidence that multi-omics AI systems are sensitive to modality-specific noise, preprocessing variation, and parameter initialization [14–18]. Reproducibility challenges persist across data reporting, computational environments, and cross-cohort evaluation, even with long-standing community standards such as MIAME, MINSEQE, and the MAQC initiatives [19, 20, 72] and more recent FAIR-aligned principles emphasizing transparency and reusability [21]. Benchmark-driven evaluation has begun to address some of these limitations [22–24], but integration of these practices across the field is still limited.

To synthesize these considerations, we introduce the TRUST framework, which outlines five components that collectively define trustworthy AI in multi-omics. Transparency emphasizes the need for interpretable model behavior supported by methods that connect predictions to biologically meaningful features, including post-hoc attribution and architecture-guided interpretability [1–7]. Reproducibility requires clear documentation, standardized reporting and stable computational workflows grounded in established data standards [19, 20], and FAIR-oriented stewardship [21]. Uniform stability calls for explicit robustness assessments through perturbation analyses, cross-cohort evaluation, and metrics designed to quantify consistency in feature selection and prediction [14–18, 55–57].

Safety and bias awareness address the risks arising from data imbalance, platform disparities, and population underrepresentation in multi-omics studies. Multi-omics datasets frequently differ in ancestry composition, sex distribution, disease subtype prevalence, and measurement platforms, creating potential sources of dataset shift and subgroup-specific performance disparities. Safety-oriented evaluation therefore requires explicit diagnostic procedures, including stratified performance analysis across demographic groups (e.g. ancestry and sex), molecular subtypes and sequencing, or assay platforms, as well as formal assessment of distributional shift between training and validation cohorts [75]. In addition, large-scale multi-omics integration studies consistently report technical heterogeneity, batch effects, and modality-specific variability as major challenges that can distort learned representations if not explicitly assessed and controlled [40–42]. Quantitative indicators such as subgroup performance gaps (e.g. ΔAUC or ΔC-index), calibration differences, and divergence measures between cohort distributions can help identify inequitable or unstable model behavior. When disparities are detected, mitigation strategies may include re-weighting schemes, stratified sampling, domain adaptation approaches designed to learn invariant representations across cohorts, adversarial training to reduce platform-specific signal leakage, or harmonization techniques to address batch and assay effects. Transparent reporting is equally critical and should include detailed documentation of cohort composition, demographic representation, assay platforms, known sampling biases, and any fairness-aware adjustments applied during model development. Established reporting frameworks such as model cards and datasheet-style dataset documentation provide structured mechanisms for disclosing such information and enhancing auditability [76, 77].

**Figure 3:**
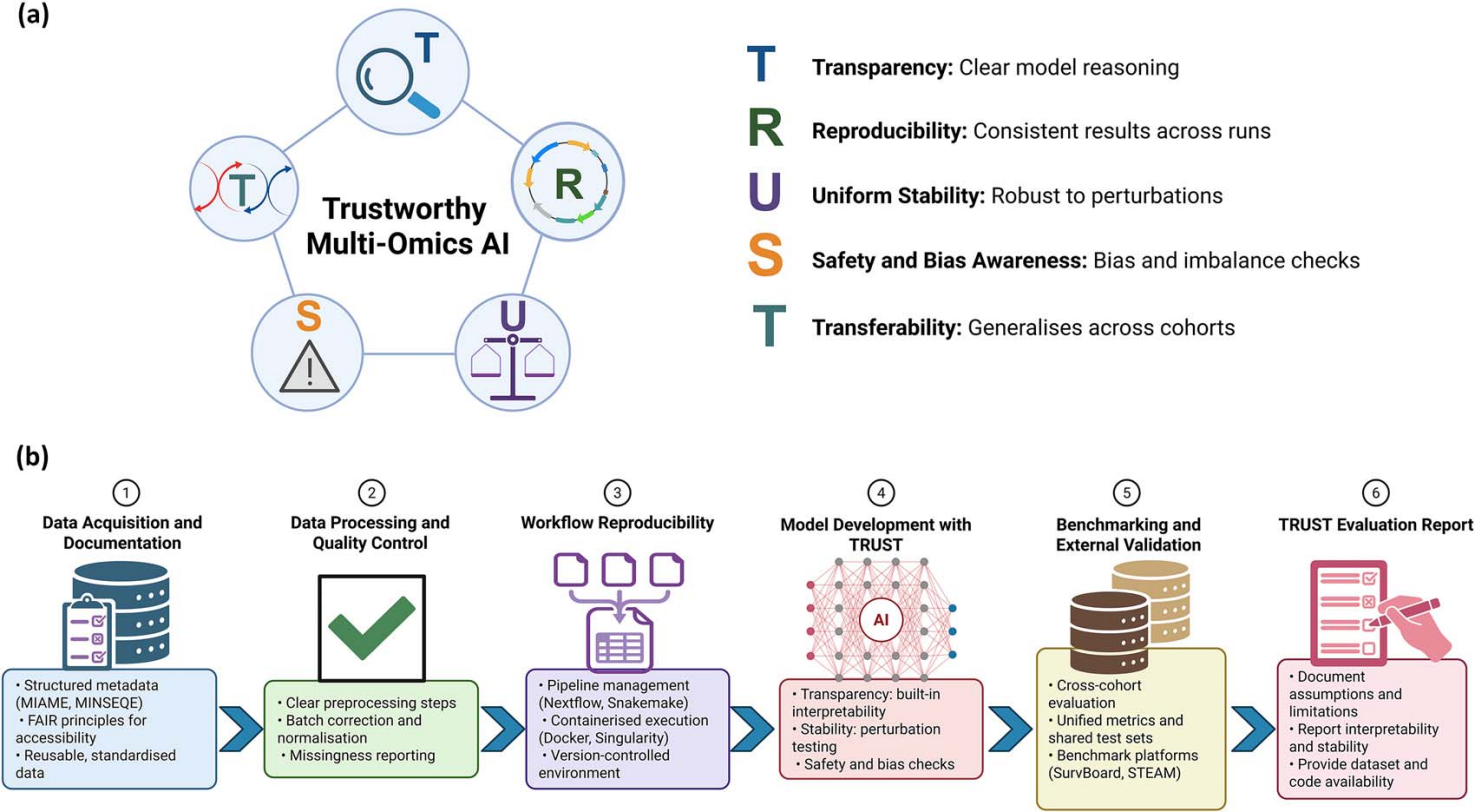
The TRUST framework and its application to multi-omics AI workflows. (a) Schematic representation of the five components of the TRUST framework: Transparency, Reproducibility, Uniform Stability, Safety and Bias Awareness, and Transferability. These components summarize the core requirements for developing trustworthy AI models in multi-omics research. (b) TRUST-aligned analytical pipeline showing practical implementation steps, including standardized metadata and reporting, reproducible workflows and containerized environments, model development with transparency and stability evaluation, and benchmark-driven assessment across datasets and preparation of TRUST-oriented model cards.

Transferability emphasizes the importance of evaluating models across datasets, platforms, and clinical or biological contexts, recognizing the strong cohort dependence reported in multiple multi-omics benchmarks [23–25]. Beyond simple external validation, transferability assessment should explicitly consider whether performance degradation is associated with demographic composition, platform differences, or shifts in molecular subtype prevalence, thereby linking generalization analysis to safety and bias diagnostics. A graphical overview of the TRUST framework and its main components is shown in Fig. 3.

Compared with traditional multi-omics AI studies, which have largely emphasized predictive accuracy within single cohorts and reported aggregate performance metrics without systematic robustness testing, the TRUST framework introduces a structured and multi-dimensional evaluation strategy. Conventional approaches often lack explicit perturbation-based stability analysis, subgroup-specific performance diagnostics, cross-platform validation, and detailed computational reporting. In contrast, TRUST formalizes these elements as core requirements, integrating stability testing, bias-aware stratification, multi-cohort benchmarking, and auditable documentation into routine model evaluation. Although this broader scope increases methodological and reporting demands, it aims to ensure that multi-omics AI systems are not only accurate but also reproducible, robust, interpretable, and generalizable across heterogeneous biological and clinical contexts.

A TRUST-aligned pipeline provides a practical blueprint for integrating these components into future methodological development. Such a workflow would begin with standardized metadata and reporting practices [19, 20, 72], followed by reproducible computational pipelines established through workflow engines and containerized environments that preserve software dependencies and execution conditions [61, 62]. Model development would incorporate transparency, stability, and reproducibility checks as routine evaluation steps, while benchmark platforms [22, 23] would support multi-dataset comparisons using unified metrics and controlled pipelines. Final outputs could be accompanied by TRUST-oriented model cards summarizing how each system performs across transparency, reproducibility, stability, bias awareness, and transferability criteria. To facilitate structured implementation and auditability of the TRUST framework, we provide a consolidated checklist mapping each component to recommended evaluation tests and reporting items (Box 1).

Box 1 **TRUST checklist for auditable multi-omics AI.**

This checklist provides a structured template for auditing and reporting TRUST-aligned multi-omics AI systems. Each component maps to minimum evaluation tests, quantitative indicators, and reporting requirements.

**T—Transparency**

Objective: Ensure model decisions are interpretable and biologically contextualized.

Required Evaluation Tests

- Application of intrinsic, post-hoc, or architecture-guided interpretability methods
- Consistency of feature attribution across runs
- Biological enrichment or pathway validation

Quantitative Indicators

- Rank correlation of feature importance across seeds
- Stability of top-k features
- Adjusted *P* values for enrichment analyses

Reporting Requirements

- Interpretability method used and rationale
- Procedure for biological validation
- Known limitations of explanation method

**R—Reproducibility**

Objective: Ensure results can be regenerated under defined data and computational conditions.

Required Evaluation Tests

- Independent rerun of full pipeline
- Cross-cohort validation where available
- Multi-dataset benchmarking

Quantitative Indicators

- Variance of performance across reruns
- Performance consistency across cohorts
- Calibration metrics

Reporting Requirements

- Data sources and accession identifiers
- Preprocessing and normalization steps
- Software versions, dependencies, and random seeds
- Public code availability

**U—Uniform Stability**

Objective: Ensure model outputs remain consistent under controlled perturbations.

Required Evaluation Tests

- ≥10 repeated trainings with different random seeds
- Bootstrap or subsampling stability analysis
- Modality dropout and graded missingness (e.g. 5%–20%)
- Noise injection proportional to feature variance

Synthetic batch perturbation

- Quantitative Indicators
- Standard deviation of predictive metrics
- Jaccard similarity or Nogueira score for feature selection
- ARI or NMI for clustering
- Performance degradation curves under perturbation

Reporting Requirements

- Number of perturbation iterations
- Magnitude of noise or masking applied
- Dispersion statistics for predictions, features, and clusters

**S—Safety and Bias Awareness**

Objective: Identify and mitigate performance disparities across data strata.

Required Evaluation Tests

- Stratified performance analysis across ancestry, sex, disease subtype, and assay platform
- Assessment of data imbalance and subgroup representation
- Formal evaluation of distributional shift between training and validation cohorts
- Cross-platform or cross-batch error analysis

Quantitative Indicators

- Subgroup performance gaps (e.g. ΔAUC, ΔC-index)
- Calibration differences across strata
- Distribution divergence metrics (e.g. KL divergence, Wasserstein distance)
- Platform-or batch-specific error rates

Mitigation Strategies (if disparities detected)

- Re-weighting or stratified sampling
- Domain adaptation or invariant representation learning
- Batch harmonization or platform correction methods
- Sensitivity analysis excluding dominant groups

Reporting Requirements

- Detailed cohort composition (ancestry, sex, subtype, platform distribution)
- Disclosure of sampling biases and imbalance
- Description of fairness-aware adjustments or harmonization steps
- Inclusion of bias-aware model card and/or datasheet-style dataset documentation

**T—Transferability**

Objective: Evaluate generalization across datasets, platforms, or biological contexts.

Required Evaluation Tests

- External validation on independent cohorts
- Cross-platform evaluation
- Domain shift analysis

Quantitative Indicators

- Performance drop between training and external datasets
- Cross-cohort calibration metrics
- Embedding similarity across domains (if applicable)

Reporting Requirements

- Description of external datasets used
- Differences in measurement platforms or preprocessing
- Interpretation of generalization gaps

Beyond methodological rigor, computational feasibility is an important consideration for multi-omics AI. Model architectures differ substantially in computational demand. Classical statistical and sparse linear models typically scale linearly with feature dimension and are computationally efficient, making them suitable for moderate-sized bulk multi-omics datasets. In contrast, deep multimodal architectures, graph neural networks, and generative models such as variational autoencoders or diffusion-based frameworks often require substantial GPU memory and training time, particularly when integrating high-dimensional single-cell, or spatial multi-omics data. Scalability challenges become more pronounced as the number of modalities, features, or samples increases, and cross-cohort benchmarking or perturbation-based stability testing further multiplies computational cost. Consequently, TRUST-aligned evaluation introduces additional resource requirements, including repeated training across seeds, resampling procedures, and modality-dropout experiments. While these demands may increase computational burden, they provide critical information regarding robustness, transferability, and reliability. Careful model selection, dimensionality reduction strategies, distributed training, and hardware-aware implementation are therefore essential for balancing methodological rigor with practical feasibility.

We situate TRUST relative to existing reporting and regulatory frameworks (Table 4).

**Table 4:** Comparison of TRUST with existing reporting, regulatory, and reproducibility frameworks.

Framework	Primary focus	What it standardizes	Coverage relative to TRUST	How TRUST extends or differs (multi-omics context)
**Model cards for model reporting** (Mitchell et al., 2019) [76]	Structured model reporting for transparency and responsible deployment	Intended use, performance metrics, evaluation data, limitations, ethical considerations	Strong alignment with transparency and partial alignment with safety and transferability; limited explicit stability requirements	Adds explicit multi-omics–specific stability testing (perturbation battery), cross-cohort benchmarking, and modality-aware diagnostics
**Datasheets for datasets** (Gebru et al., 2021) [77]	Structured dataset documentation	Data provenance, collection process, composition, recommended uses, and limitations	Strong alignment with reproducibility and safety; indirect support for transparency	Integrates dataset documentation with required evaluation diagnostics (shift testing, subgroup calibration, platform disparity assessment) and links documentation to model-level validation
**FDA/IMDRF Good Machine Learning Practice (GMLP)** [78]	Quality principles for AI/ML in medical devices	Data management, model training and evaluation processes, documentation, lifecycle governance	Broad alignment with reproducibility, safety, and transferability at principle level	Provides operational, metric-based stability, and cross-modality evaluation tailored to heterogeneous multi-omics data
**FDA AI guidance for regulatory decision-making (drugs/biologics)** [79]	Risk-based credibility assessment of AI supporting regulatory submissions	Context-of-use definition, validation expectations, documentation of model credibility	Aligns with Reproducibility and safety; limited prescriptive guidance for interpretability/stability testing in multi-omics	Specifies interpretability grounding and multi-dataset benchmarking requirements specific to omics heterogeneity and biological validation
**EMA guiding principles of good AI practice in drug development** [80]	High-level AI governance principles across medicines lifecycle	Quality assurance, transparency, traceability, risk management	Conceptual alignment across all TRUST components	Operationalizes principles through auditable checklist linking transparency, stability, bias diagnostics, and transferability to measurable criteria
**Five pillars of computational reproducibility** (Ziemann et al., 2023) [74]	Practical reproducibility of computational research	Version control, environment specification, data/code sharing, documentation	Strong alignment with reproducibility; indirect support for transparency	Extends beyond computational reproducibility to include explicit stability assessment, bias diagnostics, and cross-cohort generalization testing

Looking ahead, the principles outlined in the TRUST framework suggest several directions for advancing trustworthy multi-omics AI. Continued development of biologically grounded interpretability methods will be essential as models integrate increasingly complex transcriptomic, epigenomic, and spatial data. Robustness and consistency assessments should become a standard component of model evaluation, incorporating perturbation testing, subsampling, modality dropout, and cross-cohort analysis to ensure uniform stability across diverse datasets. Reproducibility will benefit from broader adoption of consistent metadata standards, FAIR-aligned documentation, and containerized pipelines that enable reliable reexecution across systems. Benchmark-driven development is likely to expand further, with shared datasets and standardized workflows helping address the strong dataset dependence observed in current multi-omics models. At the same time, future efforts must prioritize safety and bias awareness, ensuring equitable representation across populations, platforms, and geographic regions to mitigate disparities in data availability and clinical relevance. As multi-omics research continues to scale through single-cell, spatial, and proteogenomic technologies, the TRUST framework provides a structured foundation for guiding AI development toward systems that are transparent, reproducible, robust, and equitable, ultimately supporting safe and reliable translation into clinical practice.

## Emerging clinical applications of multi-omics AI

Several multi-omics AI systems have begun to demonstrate clinical utility in oncology and precision medicine. For example, integrative survival prediction models evaluated across TCGA cohorts have shown improved risk stratification compared with single-omics approaches, although performance variability across cancer types highlights transferability challenges [23–25]. Graph-based multi-omics classifiers such as MOGONET have reported improved patient subtype identification and biomarker discovery with external validation across independent datasets [15]. In single-cell and spatial multi-omics contexts, generative integration models have enhanced cell-type resolution and disease stratification, providing mechanistic insights that may inform therapeutic targeting [5, 53]. Despite these promising outcomes, most implementations remain at the retrospective validation stage, underscoring the need for structured stability assessment, bias diagnostics, and cross-platform benchmarking before routine clinical deployment. These examples illustrate both the translational potential of multi-omics AI and the practical importance of TRUST-aligned evaluation criteria.

## Conclusion

AI has rapidly transformed multi-omics research, enabling the integration of complex molecular layers and the discovery of biologically meaningful patterns across diverse datasets. Recent advances in foundation models and large-scale multimodal learning signal the next phase of multi-omics AI development. Transformer-based architectures pretrained on large transcriptomic, genomic, or cross-modal datasets are increasingly used to derive transferable molecular representations that can be fine-tuned across downstream tasks. Multimodal integration frameworks combining omics profiles with imaging, spatial data, and clinical variables further reflect a shift toward unified representation learning across heterogeneous biomedical domains. While these high-capacity models enhance scalability and transfer potential, they also intensify challenges related to interpretability, computational efficiency, domain shift, and bias propagation from large pretraining datasets. Ensuring that such approaches adhere to principles of stability, reproducibility, and safety will be essential for responsible clinical translation. Our bibliometric analysis shows that interest in interpretability, stability, and reproducibility has grown substantially in recent years, reflecting a broader recognition that trustworthy methodological foundations are essential for advancing multi-omics AI. Through this review, we identified key challenges and emerging opportunities across these three dimensions and highlighted the uneven adoption of best practices in current studies. The TRUST framework synthesizes these insights into a unified perspective, emphasizing transparency, reproducibility, stability, safety, and transferability as core pillars for reliable model development. Continued progress will require coordinated efforts from method developers, data generators, and the broader research community, including wider use of standardized reporting, reproducible workflows, and multi-dataset evaluation. By adopting these principles, future AI systems will be better positioned to deliver robust, interpretable, and generalizable insights that can meaningfully support biological discovery and clinical decision making.

**Key points**

- Trustworthy artificial intelligence (AI) for multi-omics requires attention to three essential pillars (interpretability, stability, and reproducibility) each of which remains inconsistently addressed in current studies.
- Our bibliometric analysis of *Briefings in Bioinformatics* (2000–2025) reveals rising emphasis on transparency, robustness, and benchmarking, especially after 2020, reflecting a shift toward more reliable multi-omics AI methods.
- Interpretability approaches in multi-omics AI now include intrinsic, post-hoc, and architecture-guided strategies, enabling deeper biological insight but still lacking standardized evaluation.
- Stability assessments are rarely implemented, despite strong evidence that multi-omics models are sensitive to modality-specific noise, preprocessing choices, model architecture, and training variation.
- We introduce the TRUST framework (Transparency, Reproducibility, Uniform Stability, Safety/Bias Awareness, and Transferability) to guide the development of robust, transparent, and clinically meaningful multi-omics AI systems.

## Supplementary Material

bbag227_Supplemental_Files

## Data Availability

No new datasets were generated or analyzed in this review article. All data discussed in this manuscript were obtained from previously published studies, publicly available repositories, or bibliographic databases cited in the text.
